# Anti-relapse activity of mirincamycin in the *Plasmodium cynomolgi* sporozoite-infected Rhesus monkey model

**DOI:** 10.1186/1475-2875-13-409

**Published:** 2014-10-17

**Authors:** Susan Fracisco, Paktiya Teja-isavadharm, Montip Gettayacamin, Jonathan Berman, Qigui Li, Victor Melendez, David Saunders, Lisa Xie, Colin Ohrt

**Affiliations:** Walter Reed Army Institute of Research, Silver Spring, MD USA; Department of Immunology and Medicine, United States Army Medical Component – Armed Forces Research Institute of Medical Sciences (USAMC-AFRIMS), Bangkok, Thailand; Department of Veterinary Medicine, United States Army Medical Component – Armed Forces Research Institute of Medical Sciences (USAMC-AFRIMS), Bangkok, Thailand

**Keywords:** Malaria, *P. vivax*, Hypnozoites, Relapse, Mirincamycin, Rhesus monkey

## Abstract

**Background:**

Mirincamycin is a close analog of the drug clindamycin used to treat *Plasmodium falciparum* blood stages. The clinical need to treat *Plasmodium vivax* dormant liver stages and prevent relapse with a drug other than primaquine led to the evaluation of mirinicamycin against liver stages in animals.

**Methods:**

*cis-*mirinicamycin and *trans*-mirinicamycin were evaluated as prophylaxis against early liver stages of *Plasmodium berghei* in mice and as antirelapse hypnozoiticides against *Plasmodium cynomolgi* in the Rhesus monkey (*Macaca mulatta*).

**Results:**

Mirincamycin was very effective against early liver stages of *P. berghei* in mice: both *cis* and *trans* enantiomers were 90-100% causally prophylactic at 3.3 mg/kg/day for 3 days orally. Both *cis* and *trans* mirincamycin, however, failed to kill dormant liver stages (hypnozoites) in the *P. cynomolgi* infected Rhesus monkey, the only preclinical hypnozoite model. Mirincamycin enantiomers at 80 mg/kg/day for 7 days orally, a dose that generated exposures comparable to that seen clinically, did not prevent relapse in any of four monkeys.

**Conclusions:**

Although efficacy against early liver stages of *P. berghei* was thought to correlate with anti-hypnozoite activity in primates, for mirincamycin, at least, there was no correlation. The negative *P. cynomolgi* hypnozoite data from Rhesus monkeys indicates that mirincamycin is unlikely to have potential as a clinical anti-relapse agent.

## Background

After more than 50 years of neglect, it is recognized that *Plasmodium vivax* infection is widespread, can be as severe as *Plasmodium falciparum* infection, and is a serious disease [1]. Treatment of *P. vivax* requires elimination of the blood stages, the cause of clinical disease, with standard blood schizonticides, and also elimination of the dormant hypnozoite stages in the liver to prevent relapse of blood infection. Primaquine is the only clinical hypnozoiticide, but its use is limited by hemolysis in Glucose-6-phosphatase deficient persons.

**Figure 1:**
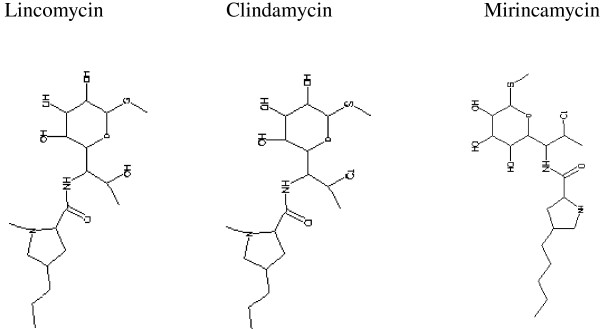
**Structures of Lincomycin (left), 7-chlorolincomycin [Clindamycin] (middle), and N-demethyl-4'-pentyl-7-chlorolincomycin [Mirincamycin] (right).**

Clindamycin is a lincosamide antibiotic which in combination with quinine is recommended for the treatment of *P. falciparum* blood stages [2]. Mirincamycin is a close chemical congener of clindamycin. (Figure 1), in mirincamycin, the nitrogen in the pyrrolidine ring is demethylated and a pentyl rather than a propyl group is attached to the 4-position. The carbon at the 4-position in the pyrrolidine ring can exist in either *cis* or *trans* configuration, resulting in either *cis*-mirincamycin or *trans*-mirincamycin [3].

The anti-malarial efficacy of mirincamycin was reported in several preclinical models between 1967 and 1972. Against *Plasmodium berghei* blood stages in mice, the 100% curative dose was the same as that for the positive control chloroquine [4]. When *P. falciparum* blood stages in Aotus monkeys were treated for seven days, mirincamycin 10 mg/kg/day was curative, clindamycin 15 mg/kg/day cured only one of three monkeys, and chloroquine 20 mg/kg/day was not curative [5]. The superior efficacy of mirincamycin *vs* clindamycin for treatment of *P. falciparum* was reiterated recently when Held *et al*. reported that mirincamycin’s *cis* and *trans* isomers are more active in vitro (IC50 = 3.2 nM and 2.6 nM) than clindamycin (IC50 = 12 nM) against *P. falciparum* clinical isolates from patients in Gabon [6].

Preclinical evaluation of liver hypnozoiticides starts with determination of prophylactic efficacy against initial infection of the liver by *P. berghei* in mice. Although *P. berghei* does not have a dormant hypnozoite stage, it is possible that prophylaxis of initial liver infection may pertain to elimination of liver hypnozoites. Compounds active in the *P. berghei* model are then tested against *Plasmodium cynomolgi* sporozoite infection in the Rhesus monkey (*Macaca mulatta*). The *P. cynomolgi* sporozoite Rhesus monkey model is the only preclinical model in which hypnozoites are consistently generated and anti-hyponozoite activity can be reasonably evaluated [7]. Mirincamycin has not been evaluated for causal prophylaxis *vs P. berghei*, but Schmidt *et al*. did investigate the prophylactic efficacy of mirincamycin in the Rhesus monkey model [8]. In a complex experiment, mirincamycin was first assessed as causal prophylaxis against initial liver infection: drug was administered at 40 mg/kg/day for 9 days surrounding sporozoite inoculation. In animals which failed prophylaxis, the same dose of drug was re-administered to assess its activity as a blood schizonticide and a liver hypnozoiticide. Re-administration of mirincamycin (40 mg/kg/day for seven days) cured the blood stages in all animals and prevented relapse in two of three animals [8].

Due to the present interest in non-haemolytic primaquine replacements, mirincamycin was investigated according to the preclinical paradigm as a causal prophylactic agent against *P. berghei* in mice followed by anti-hypnozoite activity against *P. cynomolgi* in Rhesus monkeys.

## Methods

### Causal prophylaxis in mice

Female, malaria-naïve, ICR mice were used in these experiments. On day 0, each mouse was inoculated intravenously with 100,000 *P. berghei* ANKA strain sporozoites contained in 0.1 ml of PBS with 5% bovine serum albumin. The sporozoites were obtained by dissecting the salivary glands of *Anopheles dirus* mosquitoes, which had previously been fed on donor mice infected with this *Plasmodium* strain.

Drug was administered to the mice on days -1, 0, and +1 with respect to sporozoite inoculation. Clindamycin and racemic/*cis/trans* mirincamycin were obtained from the Walter Reed Army Institute of Research (WRAIR) drug repository and dissolved in 0.5% hydroxyethylcellulose: 0.1% Tween 80. Dissolved drug was administered daily either subcutaneously (SC) or per os (PO). There were 5 animals per drug dose group.

Blood smears were obtained on days 4, 5, 6, 7, 10, 15, 21 and 31 post-sporozoite inoculation to determine parasitaemia. The day on which parasites were first seen was the day of patency. If no parasites were seen by day 31, that dose of drug was concluded to be “causally prophylactic”.

### Pharmacokinetics in mice

*Cis*-mirincamycin and *trans*-mirincamycin were administered once SC or PO at a dose of 40 mg/kg to three male mice. Blood was taken at ¼, ½, 1, 3, 6, 24, 48, and 72 hours for analysis by LC/MS and the values were subjected to non-compartmental analyses using Phoenix (version 6.1; Pharsight Corp., Mountain View, CA) to calculate pharmacokinetic parameters.

### Radical cure in Rhesus monkeys

Radical cure of *P. cynomolgi* hypnozoites in Rhesus monkeys was evaluated in the model classically used by Schmidt *et al.* [9] as recently modified [10]. *Anopheles dirus* mosquitoes were fed on a Rhesus monkey infected with *P. cynomolgi* strain B *(bastianellii*). Fourteen to 21 days after feeding, the mosquitoes were anesthetized by chilling on ice and the salivary glands were removed and lightly ground with a pestle in a solution of normal saline with 5% bovine serum albumin to free the sporozoites. Following centrifugation, the supernatant was diluted with the same solution to achieve 1 × 10^6^ sporozoites/ml ascertained by phase contrast microscopy. The salivary glands, diluent, and equipment were chilled throughout this process, and within 30-60 minutes of preparation, 1 ml of inoculum was injected into the saphenous vein of each test monkey. The monkeys were *Macaca mulatta* of Indian origin, adult (2-8 years), 2.5-8 kg, of either gender, and malaria-naïve or at least 1 yr past previous malaria-infection. Day 0 was defined as the day of *Plasmodium* sporozoite inoculation.

Drugs (*cis*-mirincamycin, *trans*-mirincamycin, chloroquine (CQ), and primaquine) dissolved in 0.5% hydroxyethylcellulose: 0.1% Tween 80 were administered to the animals daily PO via a nasogastric tube.

When parasitaemia was >5,000/mm^3^, monkeys were randomly assigned to chloroquine PO (10 mg/kg/day × 7 days) or to one of the eight experimental groups consisting of mirincamycin concurrent with chloroquine. Chloroquine, which has no direct effect on liver stages, is administered to eliminate blood stages and thus serves as a negative control in comparison with groups given anti-relapse drugs concurrently with CQ. The experimental groups received either *cis*-mirincamycin PO (20, 40, or 80 mg/kg/day), *trans*-mirincamycin PO (20, 40, or 80 mg/kg/day), *cis*-mirincamycin intravenously (IV) (20 mg/kg/day), or *trans*-mirincamycin IV (20 mg/kg/day). The IV 20 mg/kg/day dose was reduced to 5 mg/kg/day beginning on day 3 after animals developed an acute haemolytic reaction. Since these were dose-ranging experiments, the number of monkeys per group was limited to two.

Efficacy was evaluated by blood smears in which parasitaemia was assessed daily from day 7 post-sporozoite inoculation through day 21, then three times per week for 4 weeks and two times per week until parasitaemic or until day 100 post drug treatment. The day of patency was defined as the first day of slide-confirmed parasitaemia. Radical cure was defined as being without relapsing parasitaemia for 100 days post drug treatment.

### Pharmacokinetics in Rhesus

Blood was taken at 1, 2, 4, 6, 24, 48, 72, 168, and 336 hr after the 7^th^ dose of drug in the above efficacy experiments. The concentration of drug in Rhesus monkey blood was determined by LC/MS as per Khemawoot *et al.* [3]

### Animal use

The USAMC-AFRIMS Institutional Animal care and Use Committee (IACUC) and the Animal Use Review Division, U.S. Army Medical Research and Materiel Command reviewed and approved these studies. Animals were maintained in accordance with established principles under the *Guide for the Care and Use of Laboratory Animals* [NRC 1996]. The USAMC-AFRIMS animal care and use program has been accredited by Association for Assessment and Accreditation for Laboratory Animal Care (AAALAC) International.

## Results

### Causal prophylaxis in mice

Mirincamycin was much more active as a causal prophylactic agent than the lincosamide congeners clindamycin and lincomycin in the *P. berghei*-infected mouse model. When drugs were administered SC, racemic mirincamycin at 3.3 mg/kg/day (MKD) was 100% causally prophylactic in experiment 180 (Table 1), whereas clindamycin protected 3 of 5 mice at 40 MKD (Table 1: experiment 179) and lincomycin was inactive at that dose (Table 1: experiment 200). The causal efficacy of *cis* and *trans* mirincamycin administered SC was compared at 1.1 MKD and lower doses. At 1.1 MKD, the *cis*-enantiomer with five of five cures was slightly more active than the *trans*-enantiomer with three of five cures at this dose (Table 1: experiments 179, 180).

**Table 1:** **Mouse causal prophylaxis experiments**

DRUG (SC or PO)	Dose (mg/kg/day)	No. inoculated	No. 100% protected	Day of parasitaemia	Exp#
**None**	0.00	35	0	4-to-5	179
**Clindamycin (SC)**	10.00	5	0	6-to-7	179
	40.00	5	3	10,10	179
**Mirincamycin (racemate: 65% trans) (SC)**	1.10	5	3	10,10	180
	3.30	5	5	na	180
**cis-Mirincamycin (SC)**	1.10	5	5	na	180
	3.30	5	5	na	180
**trans-Mirincamycin (SC)**	1.10	5	3	10,10	179
	3.30	5	5	na	179
**Lincomycin (SC)**	1.1-to-40	5	0	4-to-5	200
**cis-Mirincamycin (SC)**	0.14	5	0	5-to-6	204
	0.28	5	0	6-to-8	204
	0.55	5	3	7,9	204
	1.10	5	4	7	204
**cis-Mirincamycin (SC)**	0.14	5	0	4-to-6	205
	0.28	5	0	6-to-8	205
	0.55	5	0	7-to-10	205
	1.10	5	3	10,10	205
**Mirincamycin (racemate: 65% trans) (PO)**	1.10	5	0	7-to-9	208
	3.30	5	3	7-to-8	208
	10.00	5	5	na	208
	40.00	5	5	na	208
**cis-Mirincamycin (PO)**	1.10	5	0	7-to-8	208
	3.30	5	5	na	208
	10.00	5	3	7-to-8	208
	40.00	5	5	na	208
**cis-Mirincamycin (PO)**	2	5	1	7--11	234
	3.3	5	5	na	234
	10	5	5	na	234
**trans-Mirincamycin (PO)**	1.10	5	0	7	206
	3.30	5	5	na	206
	10.00	5	5	na	206
	40.00	5	5	na	206
**trans-Mirincamycin (PO)**	2	5	2	7--8	234
	3.3	5	4	10	234
	10	5	5	na	234

PO dosing was investigated in experiments 206, 208, and 234 (Table 1). The enantiomers were approximately equally active (10 of 10 mice protected by the *cis-*isomer and 9 of 10 mice protected by the *trans*-isomer at 3.3 MKD) although an anomalous finding was that only three of five mice were protected by the *cis*-isomer at 10 MKD in experiment 208.

### Pharmacokinetics in mice

After 40 mg/kg once, AUC (0-infinity) was 13,952 ng*h/ml for *cis*-mirincamycin delivered SC, 7,063 ng*h/ml for *cis*-mirincamycin administered PO, 12,827 ng*h/ml for trans-mirincamycin delivered SC, and 3,093 ng*h/ml for trans-mirincamycin administered PO. Bioavailability was, therefore, 52% for the *cis* isomer and 23% for the *trans* isomer.

### Radical cure in Rhesus monkeys

Although *cis*-mirincamycin and *trans*-mirincamycin were given from 20 MKD to 80 MKD orally for 7 days, or 20 MKD for 2 days then 5 MKD IV for the next five days, no animal demonstrated radical cure (Table 2). The two control animals showed relapse within 11 to 14 days post-treatment. Except for one of the *cis*-mirincamycin 40 mg/kg animals, which is viewed as an outlier, all doses up to 80 MKD delayed relapse by at most 44 of the 100 days of follow up. Higher oral doses at least of *trans*-mirincamycin could not be given, as loose stools were seen on each day of drug administration in the 40 MKD and 80 MKD groups.

**Table 2:** **Rhesus monkey anti-hypnozoite experiments**

Drug Group	Mirincamycin dose (mg/kg/day)**	# cured monkeys/# treated monkeys	Day of parasitemic relapse	Days relapsed delayed beyond CQ group	AUC (ng*h/ml) on day 7 [mean]
Treatment of initial parasitaemia					
Chloroquine*	0	0//2	11, 14	na	na
cis-mirincamycin (PO) + CQ*	20	0//2	21, 21	8, 8	1681
cis-mirincamycin (PO) + CQ*	40	0//2	34, 75	21, 62	7125
cis-mirincamycin (PO) + CQ*	80	0//2	27, 33	14, 20	8878
trans-mirincamycin (PO) + CQ*	20	0//2	18, 18	5, 5	1343
trans-mirincamycin (PO) + CQ*	40	0//2	18, 21	5, 8	4056
trans-mirincamycin (PO) + CQ*	80	0//2	29, 44	16, 31	9080
cis-mirincamycin (IV) + CQ*	20 then 5	0//2	18, 18	5, 5	1922
trans-mirincamycin (IV) + CQ*	20 then 5	0//2	18, 21	5, 8	1789

*Chloroquine (CQ) dose was 10 mg/kg/day for 7 days per os (PO).

**Drugs given daily for 7 days.

### Pharmacokinetics in Rhesus monkeys

AUC (0-infinity) increased from approximately 1,500 ng*h/mL in animals receiving 20 MKD PO to approximately 9,000 ng*h/mL in animals receiving 80 MKD PO (Table 2). Bioavailability based on comparison of AUCs in the 5 MKD IV groups (Table 2) to the 20 MKD PO groups was 23% for *cis*-mirincamycin and 19% for *trans*-mirincamycin.

Data in healthy Rhesus monkeys administered 20 MKD PO of each isomer was recently published [3]. The ratio of AUCs in infected Rhesus monkeys in the present work compared to healthy Rhesus monkeys in Khemawoot *et al.* [3] is 66% for cis-mirinicamycin and 50% for trans-mirincamycin.

## Discussion

Mirincamycin was an effective causal prophylactic agent against *P. berghei* in mice. Both *cis* and *trans* enantiomers at a dose of 3.3 mg/kg/day for three days protected 90-100% of mice. Myrincamycin mouse causal prophylactic results were, however, unpredictive for efficacy against *P. cynomolgi* hypnozoites in Rhesus monkeys. Even mirincamycin doses of 80 mg/kg/day for seven days failed to protect a single monkey, and the day of relapse was prolonged at most by 30 days over chloroquine controls.

Pharmacokinetic analysis showed that drug exposure in Rhesus monkeys was higher than in mice and comparable to man. After 80 mg/kg/day in Rhesus monkeys, AUC was approximately 9,000 ng*h/ml. After 40 mg/kg in mice, AUC for the two enantiomers was approximately 3,000-7,000 ng*h/ml and AUC following the effective dose of 3.3 mg/kg must have been a small fraction of those values. In normal human volunteers following the 1^st^ and 29^th^ administration of the highest dose administered (375 mg), mean AUC was 3,725 ng*h/ml and 18,117 ng*h/ml, respectively [11]. The lack of efficacy in Rhesus monkeys at exposures higher than that effective in mice and comparable to that achieved clinically indicates that the negative Rhesus monkey data is not due to under dosing.

The failure of mirincamycin at 80 MKD in 4 of 4 monkeys in our hands contrasts to the success of the drug at 40 MKD in 2 of 3 monkeys in earlier work by Schmidt *et al.* [8]. Schmidt’s experimental design was to re-administer drug for anti-hypnozoite activity to parasites which had survived exposure to the same drug in causal prophylactic experiments. The parasites may have been attenuated by their prior exposure and be overly susceptible to the second administration of drug. The present anti-hypnozoite experiments evaluated efficacy when, as per the clinic, the hypnozoiticide had not previously been given to the host.

## Conclusion

Although mirincamycin as per clindamycin may well have value as a blood schizonticide, present *P. cynomolgi* Rhesus monkey experiments indicate that mirincamycin does not have value as an anti-relapse agent.
